# Toward Robust AI-Assisted Dietary Assessment for Diabetes Self-Management: Quantifying and Decomposing Large Language Model Prediction Variability From Meal Images

**DOI:** 10.2196/102715

**Published:** 2026-09-04

**Authors:** Zhaohua Wang, Daniel Lane, Kayo Waki

**Affiliations:** 1 Department of Health and Behavioral Informatics & Therapeutics (HABIT), Graduate School of Medicine, The University of Tokyo, Faculty of Medicine Bldg. 2, 2nd Floor, 7-3-1 Hongo, Bunkyo-ku, Tokyo, 113-0033, Japan, 81 3-5841-1892 ext 21892; 2 Department of Diabetes and Metabolic Diseases, The University of Tokyo Hospital, Tokyo, Japan

**Keywords:** multimodal large language models, artificial intelligence, prediction variability, dietary assessment, nutrient estimation

## Abstract

Nutrient estimation from meal images by multimodal large language models can support diabetes self-management, but its robustness is limited by variability driven by both sensitivity to visual presentation and inherent model instability. Accounting for and mitigating this variability is essential for robust AI-assisted dietary assessment.

## Introduction

Dietary assessment is fundamental to diabetes self-management. AI has advanced dietary assessment [1]. In particular, multimodal large language models (LLMs) such as the GPT family offer an automated and rapid alternative to manual approaches for estimating nutrient content from meal images [2]. The accuracy of LLMs has been validated [3], but the precision remains insufficiently investigated. Because LLMs are inherently conditional and probabilistic, their predictions inevitably exhibit variability. Low precision can reduce the accuracy of nutrient estimation and reduce patient confidence in AI-assisted self-management tools. Characterizing and addressing this variability is essential for achieving robust AI-assisted dietary assessment for diabetes self-management.

In practice, a meal can be photographed from arbitrary camera viewpoints or with arbitrary dish arrangements. Such visual variations can lead to different predictions for different images of the same meal, resulting in between-image variability. At the same time, even under deterministic settings, LLMs can still exhibit residual stochasticity [4]. Such random processes can lead to different predictions for the same image of the same meal, resulting in within-image variability. Between-image variability reflects the model’s sensitivity to visual presentation, and within-image variability reflects its internal stability. Together, they constitute the prediction variability for the same meal, reflecting the precision of LLMs.

Based on paired images with different camera viewpoints or dish arrangements, we quantified the prediction variability of GPT-4o and GPT-5.1 and decomposed it into between-image variability and within-image variability, with the aim of characterizing the precision of LLM-based nutrient estimation from meal images.

## Methods

We collected 20 meal-image pairs, each pair depicting the same meal: 10 with different camera viewpoints and 10 with different dish arrangements. We evaluated 2 multimodal LLMs, GPT-4o and GPT-5.1. For each image, we issued 60 independent single-turn requests to estimate the meal’s energy, carbohydrates, protein, fat, fiber, and salt content with fixed prompt and deterministic parameter settings (Multimedia Appendix 1). The prompt specified neither an image-parsing process nor a nutritional reference, only noting that the meal was eaten in Japan.

Let Y=μ+ε denote the nutrition content prediction, which can be decomposed into the expected prediction μ and independent stochastic noise ε with E(ε)=0. For an image pair (A,B), we define the prediction difference as $D_{A,B}=Y_{A}-Y_{B}$. The expected prediction variability on the linear scale is E. Since each request is independent, we can assume the covariance of the stochastic noises $Cov\left(\varepsilon_{A},\varepsilon_{B}\right)=0$. Consequently, we can additively decompose the expected prediction variability on a squared scale into between-image variability and within-image variability: $E\left[D_{A,B}^{2}\right]={\left(\mu_{A}-\mu_{B}\right)}^{2}+Var\left(Y_{A}\right)+Var\left(Y_{B}\right)$.

In this decomposition, $E\left[D_{A,B}^{2}\right]$ represents the prediction variability and reflects precision, ${\left(\mu_{A}-\mu_{B}\right)}^{2}$ represents the between-image variability and reflects visual sensitivity, and $Var\left(Y_{A}\right)+Var\left(Y_{B}\right)$ represents the within-image variability and reflects internal stability. To ensure interpretability, we took the square root of each variability component to convert it into a root-mean-square (RMS) magnitude on the linear scale.

For each measure, we normalized it by the pooled mean prediction of the meal $\frac{\left(\mu_{A}+\mu_{B}\right)}{2}$, to obtain the dimensionless relative magnitudes. We then derived global results by aggregating pair-level local results using bootstrap resampling, treating each image pair as a sampling unit with 10,000 iterations. Nutrient-level estimates were obtained by averaging each measure across all image pairs. Model-level estimates were obtained by averaging the pair-level mean of each measure (averaged across the 6 nutrients) across all image pairs.

## Results

**Figure 1.**
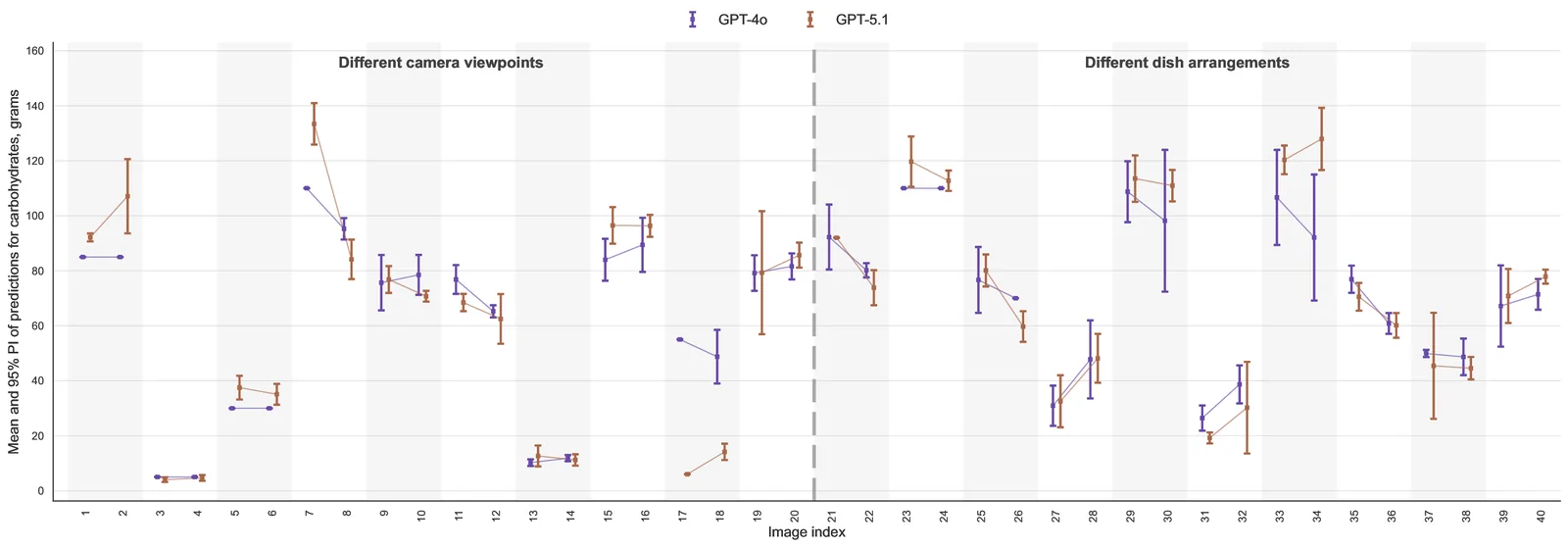
Prediction distributions for carbohydrate content of each image on GPT-4o and GPT-5.1. The plot shows the mean prediction and 95% prediction interval (PI) for carbohydrates content of each image on GPT-4o and GPT-5.1, derived from 60 repeated runs per image. Connected points indicate paired images depicting the exact same physical meal, with different visual presentation: different camera viewpoints (image pair 1 vs 2 to image pair 19 vs 20) or different dish arrangements (image pair 21 vs 22 to image pair 39 vs 40). The vertical distance between these points reflects the between-image variability, while the length of the error bars reflects the within-image variability.

Qualitative inspection revealed variability in predictions for the same meal. The dispersion of repeated predictions within one individual image evidenced within-image variability, and the difference of mean predictions between 2 paired images evidenced between-image variability (Figure 1 and Multimedia Appendix 2).

**Figure 2.**
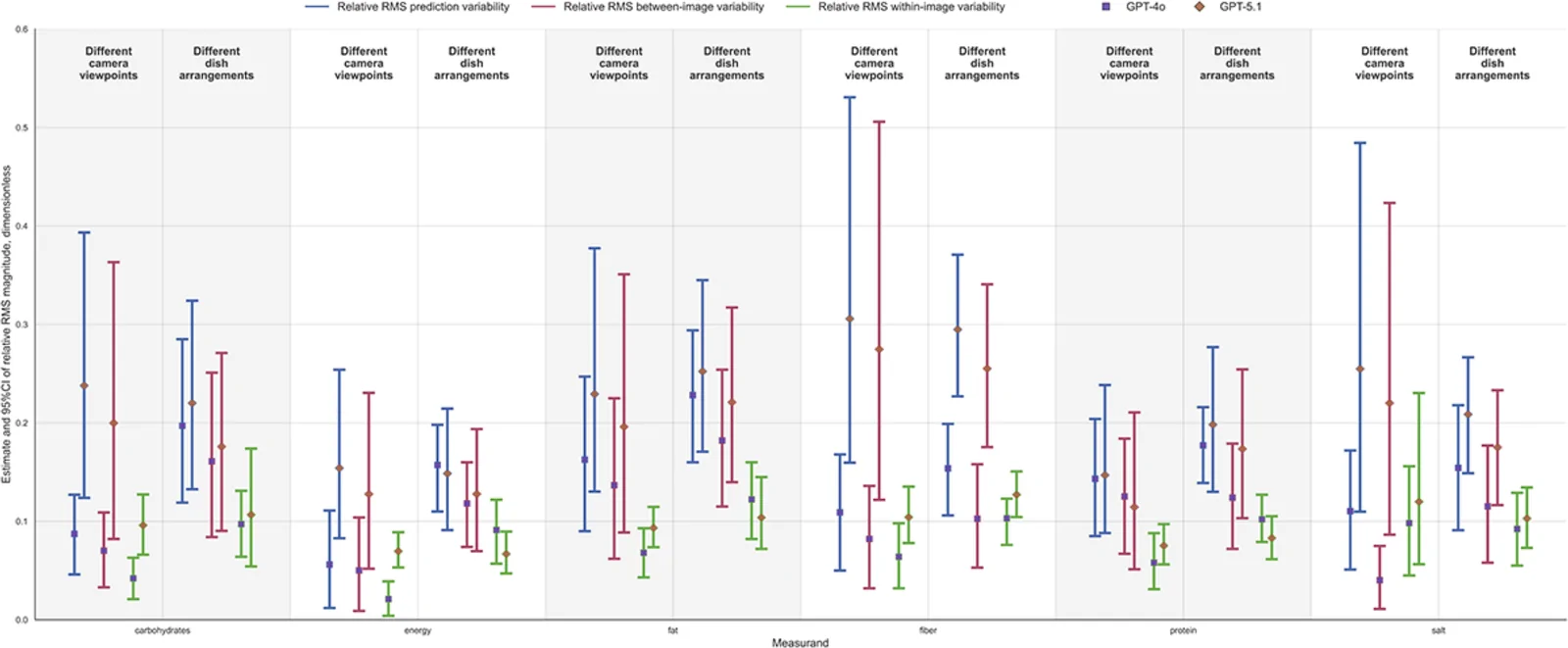
Global estimates of prediction variability and its decomposition for each measurand on GPT-4o and GPT-5.1. The plot shows the bootstrapped aggregated estimate and 95% CI of relative root-mean-square (RMS) prediction variability, relative RMS between-image variability, and relative RMS within-image variability, derived by aggregating the relative RMS magnitudes across image pairs depicting the exact same physical meal with different camera viewpoints and image pairs with different dish arrangements, respectively.

Mean absolute magnitudes directly quantified the prediction variability in natural units (Multimedia Appendix 3). Variance decomposition further quantified and decomposed the prediction variability. Regarding the measurands, both GPT-4o and GPT-5.1 showed smaller relative RMS magnitudes for energy than for the other nutrients. Among those nutrients, macronutrients such as carbohydrates and protein exhibited comparatively lower magnitudes (Figure 2). Regarding the response to image variations, GPT-4o showed higher sensitivity to dish arrangement changes than to camera viewpoint changes. In contrast, GPT-5.1 showed similarly high sensitivity to both image variations (Multimedia Appendix 4). Regarding the models, GPT-5.1 showed greater overall variability than GPT-4o. It had a higher relative RMS prediction variability, which was driven primarily by larger between-image variability, whereas its within-image variability was similar to that of GPT-4o (Multimedia Appendix 4).

## Discussion

This study qualitatively demonstrated and quantitatively assessed the prediction variability of LLMs in estimating nutrient content from meal images. There is nonnegligible prediction variability for images of the same meal, and it can be decomposed into between-image variability from visual variation of the images and within-image variability from the inherent stochasticity of the model.

Between-image variability reflects a model’s sensitivity to visual presentation. While such sensitivity is essential for capturing subtle food features, it becomes detrimental when the model overreacts to nonessential variations, such as camera viewpoints or dish arrangements. On GPT-4o, the between-image variability from dish arrangement changes was greater than that from camera viewpoint changes, as the visual variation of dish arrangement changes is often larger. On GPT-5.1, the between-image variability was higher than on GPT-4o despite its advanced architecture, which may be attributable to its increased sensitivity to subtle visual perturbations. Moreover, the between-image variability from camera viewpoint changes was similar to that from dish arrangement changes, which may also be attributable to its increased sensitivity making its response to simple camera viewpoint changes similar in intensity to its response to complex dish arrangement changes.

Within-image variability reflects a model’s internal stability. It indicates the model’s certainty about the estimation task. When estimating nutrients lacking visual cues such as salt or fiber, the model, unable to extract concrete evidence from the image, has to rely more on prior knowledge to probabilistically guess from an implicit distribution less constrained than those for energy and macronutrients, resulting in drastic fluctuations in the output for the same input.

Given the prediction variability from excessive sensitivity and internal instability, relying on a single prediction from a single image is insufficient to guarantee estimation reliability. Two pathways may mitigate the prediction variability: for a single meal, multiple photos with different camera viewpoints or dish arrangements can be captured, combining their results to limit between-image variability; for a single image, multiple requests can be issued to obtain repeated predictions, aggregating their results to mitigate within-image variability. However, these approaches should be balanced against user burden, latency and cost, and more deployment-oriented alternatives such as uncertainty-aware outputs and modular estimation pipelines require further study.

The predictions were not compared with true values, so the conclusions concern only precision, not the accuracy or validity of AI-based dietary assessment. In addition, given the inherent opacity of LLMs, the estimation process remains essentially a black box, making it difficult to address variability at the level of model processing.

## Supplementary material

10.2196/102715 Multimedia Appendix 1 Experimental models, parameters, and prompts.

10.2196/102715 Multimedia Appendix 2 Prediction distributions for energy, protein, fat, dietary fiber, and salt content of each image on GPT-4o and GPT-5.1.

10.2196/102715 Multimedia Appendix 3 Mean absolute prediction variability for each measurand and image variation type on GPT-4o and GPT-5.1.

10.2196/102715 Multimedia Appendix 4 Global estimates of prediction variability and its decomposition for each image variation types on GPT-4o and GPT-5.1.

## Abbreviations

LLM: large language model
RMS: root-mean-square
