# Supplementary material for: Toward Robust AI-Assisted Dietary Assessment for Diabetes Self-Management: Quantifying and Decomposing Large Language Model Prediction Variability From Meal Images
Source: JMIR Diabetes. 2026 Sep 4;11:e102715. doi: 10.2196/102715 (PMC13544413; doi:10.2196/102715)
Supplement: Supplementary file 1 [file diabetes-v11-e102715-s001.docx]

**Experimental models, parameters, and prompts**

Appendix 1.1 Model Versions

| Model | API identifier | Provider |
| --- | --- | --- |
| GPT-4o | gpt-4o-2024-08-06 | OpenAI |
| GPT-5.1 | gpt-5.1-2025-11-13 | OpenAI |

Appendix 1.2 API Parameters

| Parameter | Value |
| --- | --- |
| API endpoint | OpenAI Chat Completions API |
| temperature | 0 |
| top_p | 1 |
| seed | 77 |
| max_tokens | 3000 |

The seed value of 77 was arbitrarily selected prior to the experiment and used for all requests on both models, as a part of the deterministic parameter settings to control controllable sources of nondeterminism. This study focused on the residual variability that remained after fixing the seed, so additional variability introduced by variable random seed remains unknown.

Appendix 1.3 Prompts

| System prompt | You are a dietary expert assessing photographs of food eaten in Japan. Be concise in your answers. |
| --- | --- |
| User prompt | You are a dietary expert assessing photographs of food eaten in Japan. Be concise in your answers.  You are to calculate single numeric estimates (not ranges) of the nutritional content (with one digit after the decimal point and no units) of the meal or dish or drink or package of food in the photograph. If the image is not either a meal or dish or drink or package of food, or if you encounter difficulty in making estimates, respond with zeroes for all values.  Please provide a JSON object in the following format:  {   "explanation": "str", // A text explanation of the logic used in making estimates  "energy": "float", // The energy content of the meal, in kcal  "carbohydrates": "float", // The carbohydrate content of the meal, in grams  "fat": "float", // The fat content of the meal, in grams  "protein": "float", // The protein content of the meal, in grams  "salt": "float", // The salt content of the meal, in grams  "fiber": "float" // The fiber content of the meal, in grams }  Example: {  "explanation": "The meal appears to be ramen with pork topping, rice, and miso soup.",  "energy": 750,  "carbohydrates": 96.4,  "fat": 22,  "protein": 33.8,  "salt": 5.3,  "fiber": 5.2 } |
