# Supplementary material for: Toward Robust AI-Assisted Dietary Assessment for Diabetes Self-Management: Quantifying and Decomposing Large Language Model Prediction Variability From Meal Images
Source: JMIR Diabetes. 2026 Sep 4;11:e102715. doi: 10.2196/102715 (PMC13544413; doi:10.2196/102715)
Supplement: Supplementary file 2 [file diabetes-v11-e102715-s003.docx]

Mean absolute prediction variability for each measurand and image variation type on GPT-4o and GPT-5.1

| Model | Image  variations | Measurand | Mean absolute  prediction variability  [95% CI] | Relative mean absolute  prediction variability  [95% CI] |
| --- | --- | --- | --- | --- |
| GPT-4o | Different  camera  viewpoints | Carbohydrates (g) | 4.65 [1.87, 7.83] | 7.30% [3.51%, 11.22%] |
|  |  | Energy (kcal) | 25.20 [5.99, 48.33] | 5.05% [0.98%, 10.23%] |
|  |  | Fat (g) | 1.97 [0.99, 3.12] | 14.04% [6.51%, 22.65%] |
|  |  | Fiber (g) | 0.57 [0.18, 0.99] | 8.48% [3.25%, 13.97%] |
|  |  | Protein (g) | 2.90 [1.47, 4.34] | 12.49% [6.72%, 18.49%] |
|  |  | Salt (g) | 0.18 [0.06, 0.31] | 7.04% [2.78%, 11.69%] |
|  | Different  dish  arrangements | Carbohydrates (g) | 10.33 [6.67, 13.73] | 17.12% [9.60%, 25.70%] |
|  |  | Energy (kcal) | 76.13 [47.09, 105.57] | 12.32% [7.78%, 16.69%] |
|  |  | Fat (g) | 4.41 [3.10, 5.68] | 19.76% [13.44%, 26.49%] |
|  |  | Fiber (g) | 0.69 [0.36, 1.05] | 11.45% [6.87%, 16.27%] |
|  |  | Protein (g) | 4.47 [3.15, 5.75] | 15.06% [10.61%, 19.38%] |
|  |  | Salt (g) | 0.48 [0.23, 0.80] | 12.63% [6.82%, 18.85%] |
|  | Different  camera  viewpoints  or  dish  arrangements | Carbohydrates (g) | 7.50 [4.90, 10.18] | 12.24% [7.55%, 17.54%] |
|  |  | Energy (kcal) | 50.65 [29.15, 72.68] | 8.68% [5.03%, 12.38%] |
|  |  | Fat (g) | 3.19 [2.20, 4.21] | 16.90% [11.62%, 22.42%] |
|  |  | Fiber (g) | 0.63 [0.37, 0.91] | 9.99% [6.39%, 13.72%] |
|  |  | Protein (g) | 3.68 [2.64, 4.72] | 13.77% [10.00%, 17.49%] |
|  |  | Salt (g) | 0.33 [0.18, 0.52] | 9.87% [6.05%, 13.99%] |
| GPT-5.1 | Different  camera  viewpoints | Carbohydrates (g) | 10.03 [3.92, 19.49] | 21.23% [9.76%, 37.07%] |
|  |  | Energy (kcal) | 64.67 [36.38, 97.18] | 13.63% [6.40%, 23.79%] |
|  |  | Fat (g) | 4.34 [2.78, 5.81] | 21.13% [11.08%, 36.01%] |
|  |  | Fiber (g) | 1.16 [0.55, 2.06] | 27.98% [13.13%, 51.03%] |
|  |  | Protein (g) | 3.41 [2.27, 4.68] | 12.93% [7.18%, 22.14%] |
|  |  | Salt (g) | 0.42 [0.23, 0.63] | 21.93% [8.77%, 41.59%] |
|  | Different  dish  arrangements | Carbohydrates (g) | 11.23 [8.36, 14.32] | 19.72% [12.00%, 28.25%] |
|  |  | Energy (kcal) | 89.08 [53.52, 132.80] | 13.04% [7.24%, 19.71%] |
|  |  | Fat (g) | 7.44 [4.55, 11.60] | 22.93% [14.66%, 32.51%] |
|  |  | Fiber (g) | 1.24 [0.83, 1.67] | 26.13% [18.42%, 34.53%] |
|  |  | Protein (g) | 6.07 [4.02, 8.29] | 17.98% [11.14%, 26.00%] |
|  |  | Salt (g) | 0.62 [0.46, 0.76] | 18.21% [12.39%, 23.86%] |
|  | Different  camera  viewpoints  or  dish  arrangements | Carbohydrates (g) | 10.64 [6.94, 15.76] | 20.55% [13.30%, 29.53%] |
|  |  | Energy (kcal) | 77.04 [53.18, 104.18] | 13.35% [8.39%, 19.26%] |
|  |  | Fat (g) | 5.90 [4.13, 8.22] | 22.03% [14.95%, 30.71%] |
|  |  | Fiber (g) | 1.20 [0.81, 1.70] | 27.15% [18.05%, 39.33%] |
|  |  | Protein (g) | 4.75 [3.45, 6.16] | 15.47% [10.38%, 21.50%] |
|  |  | Salt (g) | 0.52 [0.39, 0.65] | 20.17% [12.61%, 30.86%] |
